# Combined habit reversal training and exposure response prevention in a group setting compared to individual training: a randomized controlled clinical trial

**DOI:** 10.1007/s00787-018-1187-z

**Published:** 2018-06-28

**Authors:** Judith B. Nissen, Martin Kaergaard, Lisbeth Laursen, Erik Parner, Per Hove Thomsen

**Affiliations:** 1 Center for Child and Adolescent Psychiatry, Aarhus University Hospital Risskov, Risskov, Denmark; 2 Section of Biostatistics, Department of Public Health, Aarhus University, Aarhus, Denmark; 3 Institute of Clinical Medicine, Health, Aarhus University, Aarhus, Denmark

**Keywords:** Tourette syndrome, Pediatric, Habit reversal training, Exposure response prevention, Group, Manual

## Abstract

Chronic tic disorders may have a huge influence on quality of life. Habit reversal training (HRT) and exposure response prevention (ERP) are effective treatments. In a blinded assessed, open trial, this study evaluates the effectiveness of a newly developed Scandinavian tic treating manual designed to treat adolescents with a chronic tic disorder, combining HRT and ERP. The study compared the efficacy of treatment based on the same manual delivered either individually or in groups. The study was an open randomized controlled clinical trial in which adolescents were randomized to either individual or group therapy. Both therapies included nine sessions. The parents were offered group-based psycho-education. The exclusion criteria were chosen to design a study that would be close to clinical practice. This is the first Scandinavian study that examines the effectiveness of a treatment manual combining HRT and ERP delivered in an individual and group setting. The study showed a significant reduction of the Total Tic score on the Yale Global Tic Severity Scale both in the individual (effect size 1.21) and group setting (effect size 1.38). A total of 66.7% of participants were considered responders. There was no statistical significant difference between the individual and group setting apart from the functional impairment score. The reductions were comparable with those shown in other studies. The participants applied both HRT and ERP, and the majority (36/59) reported an increased post-treatment experience of control. The newly designed Scandinavian manual was equally effective in the individual and group setting with effect sizes comparable with those shown in other studies.

## Background

Chronic tic disorders (CTD), including Tourette syndrome, are neuropsychiatric, neurodevelopmental disorders with a prevalence of 0.5–1%. Tourette syndrome affects mostly males (4:1) and has an age of onset between 6 and 8 years, where motor tics often precede the vocal tics [1, 2]. Though fluctuating in tic intensity, severity and localization, tic disorders may have a huge impact on quality of life and daily living [3, 4]. This places high demands on the availability of effective treatments.

European guidelines recommend a behavioral and psychosocial intervention as the primary treatment of CTD in children and adolescents [5]. Pharmacological treatment indicated by the tic disorder is viewed as a supplemental treatment [5–7]. Therapeutic treatments of CTD include habit reversal treatment (HRT) and exposure and response prevention (ERP) [5].

Habit reversal (HR) was introduced in 1973 by Azrin et al. [8] and later on as part of Comprehensive Behavioral Intervention for Tics (CBIT) [9–11]. The premonitory urge is a sensory phenomenon preceding the tic symptom and is often described as an amplifier of tics even though no relation has been shown between reductions in premonitory urge scores and treatment outcome [12] or the ability to suppress tics [13]. In HRT, a competing response is trained which helps the child to endure the internal pressure made by the premonitory urge [10, 11]. Woods et al. [11] presented a structured treatment manual describing eight sessions delivered individually over a period of 10 weeks, supplemented with three booster sessions. Two randomized controlled trials have examined the effect of HRT/CBIT compared to a control group in children/adolescent populations. Both studies showed that treatment with HRT/CBIT significantly reduced the tic intensity as a total score with effect sizes of 0.68 [14] and 0.57 [15], and as separate scores for motor and vocal symptoms, as well as impairment [14] [effect sizes of 0.49 for motor tics, 0.50 for vocal tics, and 0.57 for impairment]. A combination of HRT with mindfulness or cognitive strategies did not show additional benefit [16, 17]. Recent systematic reviews have confirmed that HRT/CBIT are effective treatments in children and adolescents with TS (SMD − 0.64, 95% CI − 0.99 to − 0.29; *n* = 133 [9, 10]). An overall moderate-to-large treatment effect of behavior therapy in reduction of symptom severity was suggested [7].

Exposure response prevention (ERP) in relation to tic disorders is described in a manual for individual treatment of children and adolescents developed by Verdellen et al. [16]. In ERP, the patient is trained to endure the premonitory urge to resist the tic symptoms. The participants are exposed to stimuli that are known to elicit tics for prolonged periods of time, and they are instructed to practice suppressing or resisting the tic symptoms. Unlike HRT, no competing response is trained. In the manual, Verdellen et al. [18] described 12 structured sessions using ERP. Furthermore, the manual described ten sessions of HRT. In a single randomized controlled study, it was shown that both treatment modalities resulted in a significant effect measured by YGTSS, tic frequency counted in the institute and at home. Effect sizes of 1.42 and 1.06 were comparable for ERP and HR, respectively [19].

Both manuals for HRT/CBIT and ERP [11, 18] describe structured sessions focusing on either HRT or ERP for individual therapy.

Group therapy has been shown to be effective in relation to other psychiatric diagnoses, which is not least known from cool kids sessions in relation to anxiety disorders in children and adolescents [20]. Children and adolescents often ask for contact to peers with comparable problems. Only one study has examined the effect of HRT in a group setting compared with group educational therapy [21]. Both group interventions showed a positive effect. However, the reduction of motor tics was larger in the HRT group. Still, a direct comparison of the effect of individual treatment compared to group therapy is lacking.

Furthermore, children and adolescents with CTD differ not least in relation to the occurrence of comorbid conditions, where almost 90% experience another psychiatric or somatic condition [22, 23]. Thus, it is still unknown whether age, tic severity, and comorbid conditions may alter or influence a child’s ability to engage in therapy or may influence the preference of one therapy setting over another. Clinical effectiveness of a combined treatment offering training in both HRT and ERP is still unknown.

The aim of this blindly assessed open trial was to evaluate the effectiveness of a newly designed Scandinavian manual combining HRT and ERP [24]. The study was designed to compare the efficacy of therapeutic treatment based on the same manual, delivered individually or in a group setting. The effectiveness was compared with the treatment results shown in other studies.

## Methods

### Design

The study was an open randomized controlled clinical trial for children and adolescents with Tourette syndrome or chronic motor or vocal tic disorder. The participants were randomized to either individual training or group therapy. Both settings included eight sessions and an additional 9th booster session. In the individual setting, the parents participated in the last 15 min of each session. In the group setting, the parents participated in the group at the end of the second, fourth, eighth, and ninth session. Furthermore, the parents were offered a group-based 2-h session of psycho-education.

The study was implemented at the specialized Tourette outpatient clinic at CAP, Risskov, Aarhus. All therapists were qualified psychotherapists trained in HRT and ERP. The therapists were offered supervision.

### Participants

**Fig. 1:**
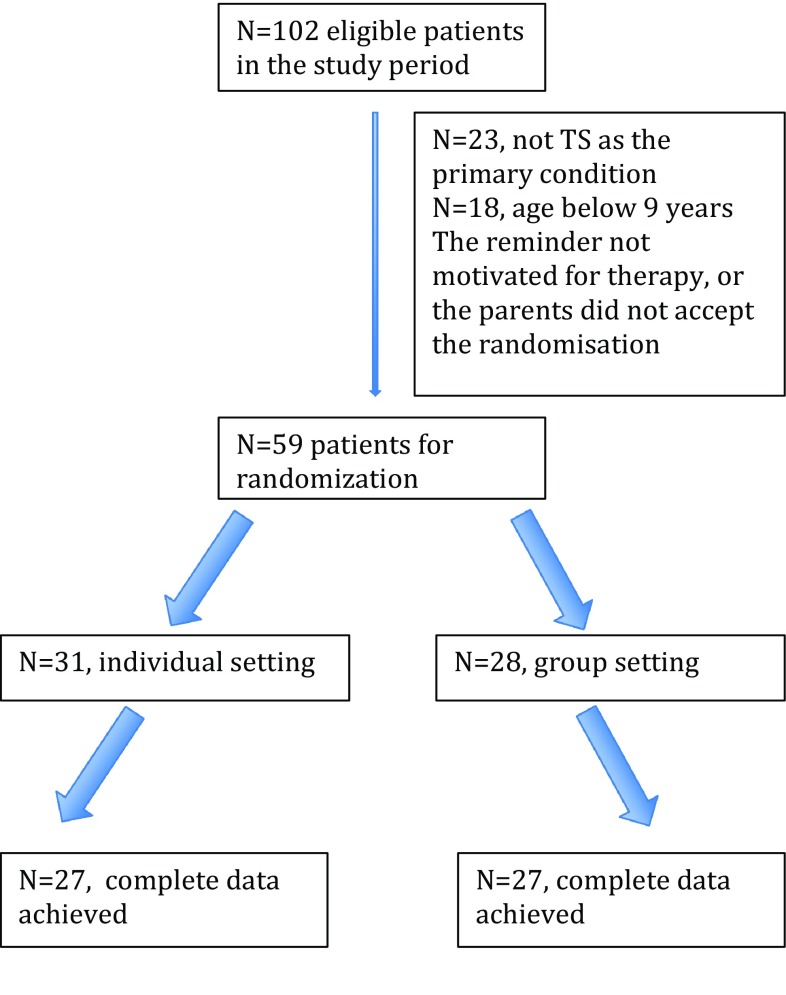
Flowchart for inclusion and exclusion of participants in the tic treatment study

Inclusion started in November/December 2015 and was concluded in September 2017. Eligible participants were children and adolescents aged 9–17 years with a primary diagnosis of either Tourette syndrome or a chronic motor/vocal tics disorder according to the WHO ICD-10 diagnostic criteria and the Diagnostic and Statistical Manual of Mental Disorders, Fourth edition, Text Revision, and of moderate or greater severity corresponding to a total score on the Yale Global Tic Severity Scale (YGTSS) [25, 26] higher than 13 (higher than 9 if only motor or vocal tics were described) [14] (Fig. 1). The inclusion and exclusion criteria were chosen as to ensure that the study would be as close to the clinical practice as possible. Thus, exclusion criteria included disorders that required immediate treatment: psychotic disorder, primary severe depression, suicidal ideation or attempts, primary severe anorexia nervosa. Furthermore, children and adolescents were excluded if their IQ was below 70, they had a lifetime diagnosis of pervasive developmental disorder, or if they had been treated with HRT or ERP during the last 6 months. Patients were included in the study if sufficient treatment had been offered for a comorbid condition, including depression, severe ADHD or anorexia nervosa, and where the tics symptoms still met the inclusion criteria. Children and adolescents on psychotropic medications for tics or other psychiatric disorders were included if medication was stable with no planned changes during the therapy period.

### Assessment methods and outcome measures

Diagnostic eligibility was established using a modified version of the Schedule for Affective Disorders and Schizophrenia for School-Age Children—Present and Lifetime version (K-SADS-PL) administered to the parents and child/adolescent separately. The K-SADS-PL information was used to confirm a primary diagnosis of chronic tic disorder and to ensure that none of the exclusion criteria were met. Furthermore, the parents were asked to provide background information and to rate the CBCL, SDQ, and the sensory profile. The parents and the child/adolescent were asked to provide specific information concerning the tic disorder. An overview of the diagnostic instruments is provided in Table 1. There were no significant in-between differences in severity scores between time of primary assessment and start of treatment (baseline).

**Table 1:** An overview of the scales and instruments used at baseline and for evaluating treatment outcome

Instrument	Assessment/baseline	Treatment outcome	Time of evaluation Session number
Diagnostics instruments	K-SADS [20]		
	CBCL [26]		
	Background information		
	Sensory profile [27]		
Severity score	YGTSS [23, 24]	YGTSS	8th, 9th, FU
	Global assessment scale	Global assessment scale	8th, 9th
Other instruments	SCARED [28–30]	SCARED	4th, 8th, 9th, FU
	MFQ [31–33]	MFQ	4th, 8th, 9th, FU
	Premonitory Urge Scale [34]	Premonitory Urge Scale	4th, 8th, 9th, FU
	Beliefs-scale [35]	Beliefs-scale	4th, 8th, 9th, FU
		Questions concerning the preferred method and best quality outcome	8th

Primary outcome measures were the Yale Global Tics Severity Scale (YGTSS) at session 8. Furthermore, the child/adolescent and the parents were asked to assess the global severity of tics on a 0–10 severity scale. Secondary outcome measures included Screen for Child Anxiety Related Emotional Disorders (SCARED), the Mood and Feelings Questionnaire (MFQ). Finally, the child/adolescent completed the Premonitory Urge Scale (PUTS) and Beliefs About Tics Scale (BATS) for analysis of the importance of the premonitory urge and the thoughts and beliefs about tics. An overview of the scales is shown in Table 1.

#### Schedule for Affective Disorders and Schizophrenia for School-Age Children—Present and Lifetime version (K-SADS-PL)

A modified version of the Schedule for Affective Disorders and Schizophrenia for School-Age Children—Present and Lifetime version (K-SADS-PL). The K-SADS-PL is a semi-structured diagnostic interview examining a range of child psychopathology (age 7–17 years). In the present study, screening symptoms and supplemental symptoms were used for selected diagnoses including depression, anorexia nervosa, ADHD, psychosis, OCD, and anxiety disorders. The interview was conducted with both the parents and the patients. In the present study, diagnoses of tic disorder or comorbidity were based on symptoms classified as certain only. The K-SADS-PL has shown a good inter-rater reliability [27].

#### Child Behavior Checklist (CBCL)

CBCL is a parent questionnaire evaluating a range of behavioral and emotional problems in children and adolescents. The questionnaire is used in the age range 6–18 years. CBCL has 113 items rated on a three-point scale (0 = not true; 1 = sometimes true; and 2 = often true). The results are depicted both in a total problem scale and several subscales. Test–retest reliability has been reported as 0.95–1.00, and the internal consistency as good to excellent [28].

#### Background information

As part of the general assessment at baseline, parents were asked questions covering occupation and educational background as well as questions aimed at identifying the presence of parental psychopathology and any family history of tic or other psychiatric and/or somatic disorders. Furthermore, both the patients and the parents were asked questions aimed at determining the age of onset and describing preceding, reducing and exacerbating factors, as well as the general course of the disorder. Finally, the parents and the patients were asked to describe the personal characteristics of the child.

#### Sensory profile (SP)

SP is a collection of questionnaires for different age groups The aim of the questionnaires is to assess children’s responses to commonly occurring sensory events and to evaluate the ability to process the sensorimotor impressions. SP includes 125 questions grouped into three main areas: sensory processing, sensory modulation and behavior, and emotional response. SP is standardized in the USA, using 1037 children with and without difficulties. The validity and reliability of SP are acceptable [29].

#### Yale Global Tics Severity Scale (YGTSS)

The YGTSS is a clinician-administered semi-structured interview including a checklist of all tics present in the past week. The YGTSS severity rating covers five dimensions divided into ten items including the number of tics, frequency, intensity, complexity and interference of the motor and vocal tics, and a separate evaluation of the functional impairment. The scores are summed to yield separate motor and vocal tic scores (0–25) and a combined total tic score (0–50). The functional impairment scale (range 0–50) is assessed, rating the tic-related disability over the past week. YGTSS has been shown to have high internal consistency and stability [25, 26].

#### Global assessment scale

The global assessment scale was developed for the present study and is a Likert scale (0: no symptoms–10: maximum symptoms), where the child/adolescent makes a global assessment of the severity of the tic disorder, the frequency of the tic symptoms, and the intensity of the premonitory urge. The child/adolescent was asked to assess at baseline, and at session 8.

The same scale was used at every visit to evaluate the severity of every single tic symptom (the severity of the tic, the frequency of the tic, and the preceding premonitory urge of the tics)

#### Screen for Child Anxiety Related Emotional Disorders (SCARED)

SCARED includes separate versions for parents and the child/adolescent. It includes 41 items rated on a three-point scale and assesses the occurrence of anxiety symptoms based on DSM-IV. Scores range from 0 to 82. The questionnaire is validated in children and adolescents (age 8–18 years), and the internal consistency has proven excellent [30–32].

#### The Mood and Feelings Questionnaire (MFQ)

MFQ assesses the occurrence of depressive symptoms, using 13 items rated on a three-point scale [33]. Scores range between 0 and 26, where high scores indicate a severe functional impairment [34]. The scale is prepared in separate versions for children/adolescents (age 8–18 years) and parents. The internal consistency is good [35].

#### Premonitory Urge Scale (PUTS)

PUTS is a short self-reporting scale with nine items prepared to measure the tic-related premonitory urge. The scale was developed by D. Woods, USA. The scale has proven consistent and with a high stability [36]. The scale was translated into Danish by the principal investigator (J. Nissen). After a re-translation into English, the scale was approved by D. Woods, USA.

#### Beliefs About Tics Scale (BATS)

BATS is a self-reporting scale with 20 items developed to assess the different beliefs children and adolescent experience in relation to tic symptoms and to suppressing the tic symptoms. The scale was developed by A. Apter, Israel. Studies have shown a high validity in relation to the experience of premonitory urge (PUTS) and to the severity and functional impairment related to tics (YGTSS). The scale has shown a high internal consistency [37]. The scale was translated into Danish by the principal investigator (J. Nissen). After a re-translation to English, the scale was approved by Dr. Steinberg, Schneider Children’s Medical Center, Israel.

#### Quality questions

The child/adolescent is asked to answer questions concerning which methods they prefer (HRT and/or ERP) and which changes they want to emphasize as the most important (effect on mood, tic intensity and impairment, the belief of being able to control tics, attention, conflicts with others, and/or self-esteem).

### Randomization and blinding

Randomization was performed in relation to the clinical conference where a diagnosis of a chronic tic disorder was confirmed. Furthermore, the exclusion criteria were evaluated. Randomization was performed using a stratified block randomization where every fourth diagnosed patient alternately directed the following three patients to either individual or group treatment. This procedure was chosen to ensure as short a latency period as possible.

Evaluations of treatment response were made by an independent evaluator who was not blinded to the treatment allocation, yet not involved in the treatment of the patient, and blinded to any previous evaluations. The evaluators were a specialized psychologist and a child and adolescent psychiatrist with several years of experience in diagnosing, evaluating and treating tic disorders. A random sample of 18 samples were audiotaped (10%) and evaluated by another rater with extensive experience and expertise in the use of the YGTSS. The analysis revealed that the intraclass correlation coefficient was 0.88 (95% CI 0.72–0.95) for Total Tics score (motor and vocal tics), and 0.89 (95% CI 0.74–0.95) for functional impairment.

### Treatments

The therapeutic treatment for individual and group setting was based on a newly developed manual [22] adapted by the individual treatment manuals by Woods et al. [11] and Verdellen et al. [18]. The newly designed manual described a nine-session therapy (eight sessions and a booster session) for either individual or group treatment. An overview of the treatment sessions is presented in Table 2. All participants trained in HRT for two sessions (session 2 and 3) and in ERP for two sessions (session 4 and 5). In the following sessions, the participants were trained in both treatment modalities depending on the presented symptoms. Thus, if the child for instance was bothered with a certain tic at school, a competing response could be selected and trained for that particular tic. For the remainder tic symptoms, ERP would be trained. The content of the sessions was similar regarding individual treatment and group-based treatment whereby outcome measures were comparable.

**Table 2:** An overview of the sessions

Session 1	Psycho-education about tic disorders
Session 2	Introduction and training in HRT
Session 3	HRT continued. How to tell others about tics
Session 4	ERP introduction and training
Session 5	ERP continued. Comorbidity
Session 6	HRT and/or ERP training
Session 7	HRT and/or ERP training. Quiz: what do you now know about tics?
Session 8	HRT and/or ERP training. Relapse prevention
Session 9	HRT and/or ERP training. Relapse prevention. How to meet a new tic

### Sessions

All sessions consisted of a review of homework, of tic reducing or exaggeration factors or situations, review of tic symptoms and severity, premonitory urge intensity, review of the applicable strategies to combat the tic symptoms and to control premonitory urge, and the goals seen in relation to the homework. At the end of the session, homework was assigned for the child/adolescent, which also included a discussion of the parent’s role in the training program to define the home assignment for the parents.

### HRT condition

Each HRT session included a review of the specific tic symptoms with a detailed description of the premonitory urge, the localization, and the course of the urge and the tic symptom. HRT included awareness training, competing response definition, detailed description and training, and social support. The therapist-assisted practice was a key component of the HRT training.

### ERP condition

Each ERP session included a review of all tic symptoms with a detailed description of the premonitory urge. ERP training included awareness training, including the training of “just” looking at the premonitory urge, calmly and softly describing the localization, the travel through the body, and the intensity of the feeling. The child/adolescent was encouraged to describe how they could visualize the feeling in the body. Again, the therapist-assisted practice in ERP training was a key component.

### The group session

The groups consisted of four participants and one therapist. Every session lasted 2 h and the parents participated in 20 min at the end of session two, four, eight, and nine.

### The individual treatment

Every session lasted 1 h and the parents participated 15 min at the end of every session.

### Parent teaching

All parents were invited to participate in a 2-h teaching and psycho-education held in groups with 10–20 participants. The focus for the teaching was

1. What is a tic disorder?
2. How to understand the occurrence of tics and the phenotypic presentation of tics.
3. Function-based assessment and intervention.
4. Psychosocial and therapeutic interventions with special focus on HRT and ERP.
5. Training at home—how can the parent support and stimulate the training?

### Statistical analysis

Primary outcome measures included the YGTSS subscores and total score. Furthermore, the global assessment of severity, frequency, and premonitory urge was examined.

Effect sizes from the present study were compared with results from both individual and group treatment studies. The improvement at post-treatment assessment was tested in each group using a paired *t* test. The effect sizes were calculated by a ratio of the mean difference and the standard deviation of the difference between baseline and follow-up (SD diff). Confidence interval (CI) of the effect sizes was computed using the non-parametric bootstrap with 100 replications and normal-based standard error. Based on the study by Verdellen et al. [19], a combined effect size for ER and HRT was estimated as 1.24 and a combined ratio between the standard deviation of the difference between baseline (SD_diff_) and follow-up, and the pooled standard deviation of baseline and follow-up SD_within_ (SD_diff_/SD_within_) was estimated as 0.64. Using a study sample size of 60 (30 persons in each group), we expected to estimate an effect size in each group with a 95% confidence interval given by ± 0.23.

The efficacy improvements were compared between the two treatment groups, using the unpaired *t* test. The occurrence of systematic differences was evaluated by the average difference and confidence intervals. The random differences were quantified by the limit of agreement (95% predicted intervals). The agreement between two raters was accessed using the intraclass correlation coefficient (ICC).

Baseline characteristics and pre-treatment TS severity scores were compared between the groups, using univariate Chi test for categorical variables and *t* tests for continuous variables.

Differences in TS scores were checked with respect to the assumptions of normality using the normality plot and homogeneity of variance using the *F* test. Significance was defined as a *p* value of less than 0.05.

## Results

### Baseline characteristics

102 adolescents were screened and 59 were randomly assigned to treatment in either an individual setting or group setting (Fig. 1). The age of the participants ranged from 9 to 17 years (mean = 12.24, SD = 2.32) and *N* = 37 (62.7%) were males. A total of *N* = 25 (42.4%) were treated with medication, including methylphenidate (*N* = 10), atomoxetine (*N* < 5), neuroleptics (*N* < 5), antidepressant (*N* < 5) and melatonin (*N* = 5). Baseline characteristics are shown in Table 3. There were no statistical between-group differences in relation to baseline severity scores, parental and child scores for SCARED or MFQ, CBCL scores, or to the description of premonitory urge. However, individual participants reported a significantly increased tendency to experience a not-just-right sensation, and reported an increased number of OC symptoms. The most often reported OC symptoms were ritualized behaviors associated with magic, order and symmetry, repeating behavior, and counting (66%). The content of OC symptoms was comparable for the two treatment groups. Furthermore, there was a significantly larger number of participants with ADHD in the groups (42.9%) compared to the individual treatment (25.8%). Attrition in the group setting was 1/28 and individual setting 4/31. A total of *N* = 49 (83.1%) reported a lifetime increased sensitivity, of which the majority reported tactile hypersensitivity (*N* = 29, 59.2%) and increased sensitivity to noise and sounds (*N* = 35, 71.4%). *N* = 28 reported to have no strategies to work against their tics. *N* = 26 had experience in suppressing their tics at school, but experienced an increased intensity when returning to their homes.

**Table 3:** Baseline characteristic

	Individual setting (*N* = 31)	Group setting (*N* = 28)	Total group (*N* = 59)	*p*
Age	12.30 (2.63)	12.18 (1.96)	12.24 (2.32)	0.85
Gender (male)	18 (58.1%)	19 (67.9%)	37 (62.7%)	0.45
Baseline YGTSS				
Total score	51.52 (13.04)	48 (12.12)	49.85 (12.62)	0.29
Motor score	15.03 (3.57)	15.25 (3.33)	15.14 (3.43)	0.81
Vocal score	9.16 (4.89)	8.43 (5.20)	8.81 (5.01)	0.58
Total Tic score	24.19 (6.94)	23.68 (6.78)	23.95 (6.81)	0.77
Functional impairment	27.39 (7.74)	24.25 (8.04)	25.90 (7.98)	0.13
SCARED				
Score by parents (*N* = 56)	21.04 (10.98)	22.71 (18.17)	21.88 (14.90)	0.68
Score by children (*N* = 52)	22.61 (11.81)	26.08 (17.16)	24.21 (14.48)	0.39
MFQ				
Score by parents (*N* = 56)	5.79 (5.19)	5.75 (5.41)	5.77 (5.25)	0.98
Score by children (*N* = 53)	4.36 (3.91)	5.12 (3.82)	4.72 (3.85)	0.48
Occurrence of not-just-right [*N* (%)]	26/31 (83.9%)	15/28 (53.6%)	41/59 (69.5%)	0.018*
Occurrence of OC symptoms [*N* (%)]	26/31 (83.9%)	16/28 (57.1%)	42/50 (71.2%)	0.024*
Occurrence of premonitory urge [*N* (%)]	29/31 (93.5%)	25/28 (89.3%)	54/59 (91.5%	0.20
CBCL total score (*N* = 58)	39.27 (25.39)	41.44 (25.96)	40.30 (25.46)	0.75
CBCL score 1	10.43 (8.99)	12.11 (9.12)	11.24 (9.01)	0.48
CBCL score 2	12.93 (9.15)	12.46 (10.07)	12.71 (9.52)	0.85
CBCL score 3	5.33 (4.20)	5.07 (4.88)	5.21 (4.50)	0.83
CBCL score4	7.57 (5.02)	6.96 (4.22)	7.28 (4.62)	0.62
CBCL score 5	3.07 (3.14)	3.75 (3.11)	3.40 (3.12)	0.41
CBCL score 6	3.2 (2.64)	3.18 (2.88)	3.19 (2.74)	0.98
CBCL score 7	2.2 (2.76)	3.11 (2.91)	2.64 (2.85)	0.23
Diagnosis of anxiety disorder [*N* (%)]	6/31 (19.4%)	4/28 (33.6%)	10/59 (16.9%)	0.53
Diagnosis of ADHD [*N* (%)]	8/31 (25.8%)	12/28 (42.9%)	20/29 (33.9%)	0.038*
Diagnosis of depression [*N* (%)]	1/31 (3%)	0/28 (0%)	1/59 (1.7%)	0.35
Problems with structure and planning [*N* (%)] (ICD-10:DF83.9)	3/31 (9.7%)	4/28 (14.3%)	7/59 (11.9%)	0.60

Total Tic score = sum motor score and vocal score, total = sum Total Tic score and functional impairment

*Significant difference between individual setting and group setting *p* < 0.05

### Outcome

**Fig. 2:**
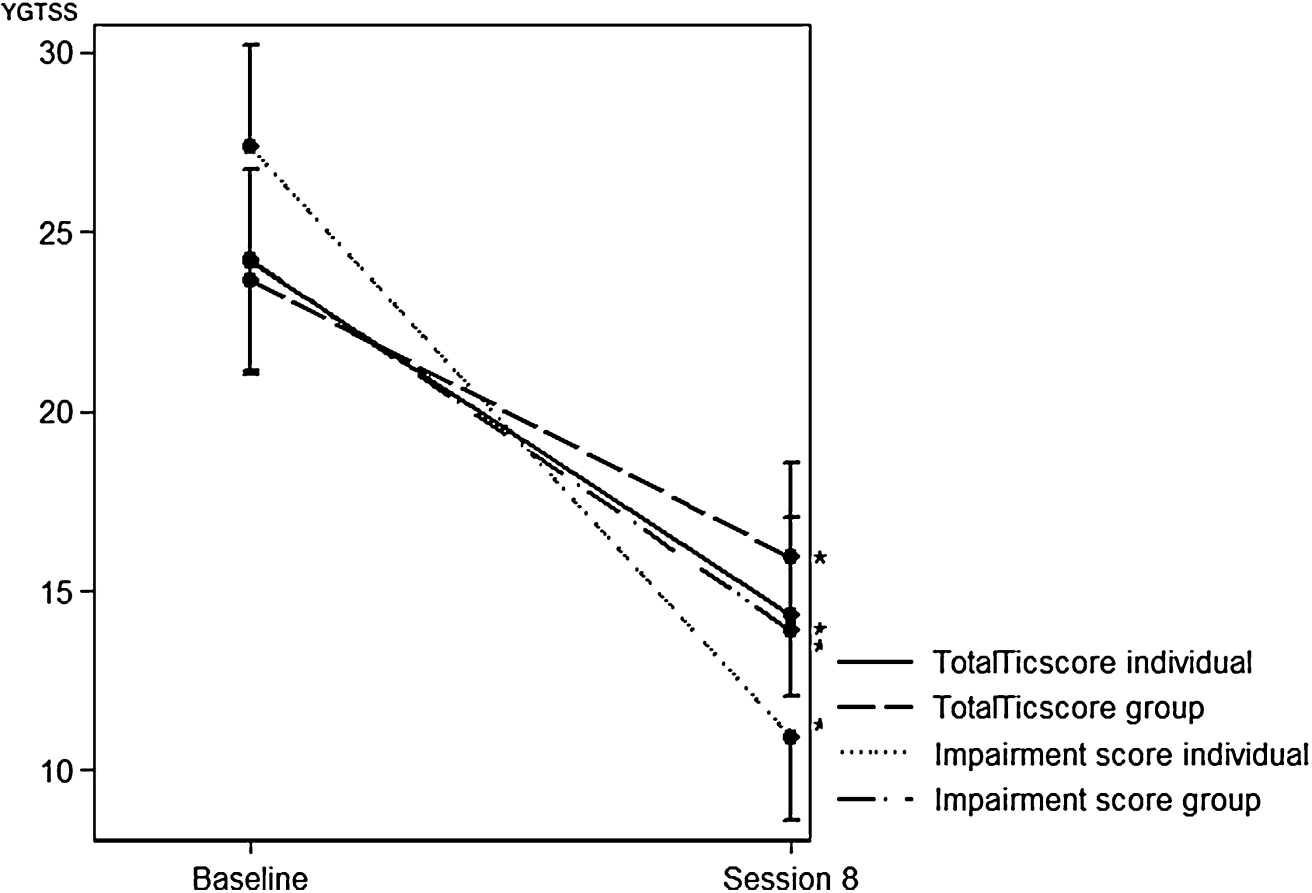
Total Tic score and functional impairment score from baseline to eighth session for each of individual therapy and group setting. *p < 0.05 significant score reductions from baseline to eighth session. *YGTSS* Yale Global Tics Severity Scale

Mean scores, effect sizes, and confidence intervals on the difference between individual and group setting for all study outcomes are shown in Table 4. After eight sessions, there was a decrease in the Yale Global Tics Severity total tic score of 9.48 (SD = 7.84) points for individual therapy and 7.48 (SD = 5.44) points for group therapy showing effect sizes of 1.21 (0.79–1.63) and 1.38 (0.84–1.64), respectively. Defining a responder level at 25% reduction of YGTSS total tic score [38], 66.7% in both settings would be considered as responders (Table 4). There were no between-group differences (individual versus group treatment), in total scores, motor scores, vocal scores, or in Total Tic scores on the YGTSS. For the functional impairment score, a significantly greater reduction was shown for the individual treatment (*p* = 0.025) (Fig. 2). Likewise, the reductions in global frequency and global stress were rated greater in the individual treatment compared to group treatment.

**Table 4:** Outcome after eight sessions

	Pre [mean (SD)]	Post [mean (SD)]	Diff [mean (SD)]	Effect size (± SD)	PPI > 25% (%)	*p*
Individual total	50.89 (12.46)	25.59 (10.04)	25.30 (15.24)	1.66 (1.11–2.21)	81.5	< 0.0001*
Individual motor	14.63 (3.56)	9.81 (3.44)	4.81 (4.10)	1.18 (0.71–1.64)	44.4	< 0.0001*
Individual vocal	9.15 (4.44)	4.48 (4.04)	4.67 (4.64)	1.01 (0.62–1.39)	63.0	< 0.0001*
Individual Total Tic score	23.78 (6.53)	14.30 (5.62)	9.48 (7.84)	1.21 (0.79–1.63)	66.7	< 0.0001*
Individual Functional impairment	27.19 (7.78)	10.93 (5.89)	16.26 (9.77)	1.66 (1.00–2.33)	92.6	<0.0001*
Group total	47.89 (12.33)	29.93 (13.33)	17.96 (11.34)	1.58 (0.99–2.18)	74.1	< 0.0001*
Group motor	15.22 (3.39)	10.52 (4.34)	4.70 (3.78)	1.24 (0.73–1.76)	48.1	< 0.0001*
Group vocal	8.19 (5.13)	5.41 (4.25)	2.78 (3.90)	0.71 (0.26–1.17)	51.9	0.001*
Group Total Tic score	23.41 (6.75)	15.93 (6.66)	7.48 (5.44)	1.38 (0.84–1.64)	66.7	< 0.0001*
Group functional impairment	24.41 (8.15)	13.89 (8.01)	10.52 (8.48)	1.24 (0.84–1.91)	70.4	<0.0001*
Total group total	49.39 (12.37)	27.76 (11.89)	21.63 (13.81)	1.57 (1.19–1.94)	77.8	< 0.0001*
Total group motor	14.93 (3.46)	10.17 (3.90)	4.76 (3.90)	1.22 (0.94–1.49)	46.3	< 0.0001*
Total group vocal	8.67 (4.77)	4.94 (4.14)	3.72 (4.35)	0.86 (0.58–1.13)	57.4	< 0.0001*
Total group Total Tic score	23.59 (6.58)	15.11 (6.16)	8.48 (6.76)	1.26 (0.94–1.57)	66.7	<0.0001*
Total group functional impairment	25.80 (8.01)	12.41 (7.12)	13.39 (9.51)	1.41 (1.00–1.82)	81.5	< 0.0001*
Global impairment	7.31 (1.55)	4.26 (2.30)	3.05 (2.15)			< 0.0001*
Global frequency	7.23 (1.78)	4.46 (2.38)	2.77 (3.05)			< 0.0001*
Global stress	6.85 (1.90)	3.51 (2.21)	3.33 (2.26)			< 0.0001*
Global urge	5.44 (3.10)	3.85 (2.58)	1.59 (3.81)			0.013*

Completer sample (*N* = 54). Means and differences (pre- and post-treatment scores) (SD), effect sizes, and the percentages of patients who improved > 25% (PPI > 25%). Total Tic score = sum motor score and vocal score, total = sum Total Tic score and functional impairment

*Significance *p* < 0.05

**Fig. 3:**
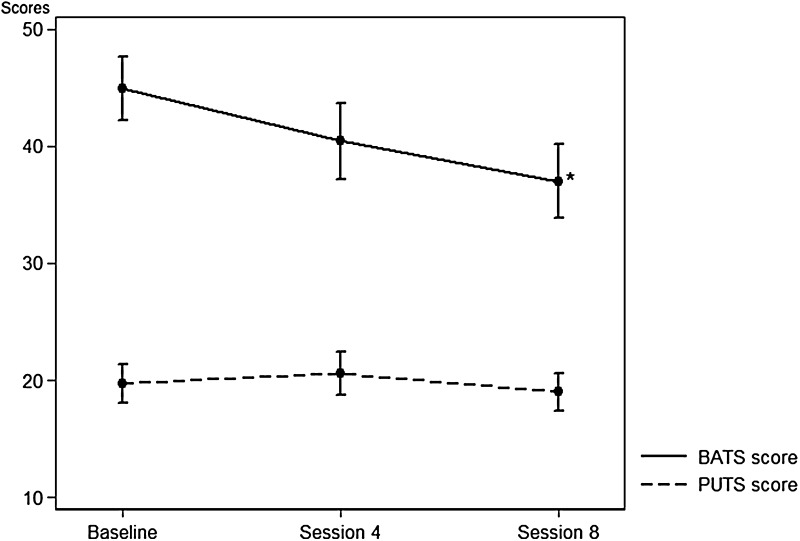
PUTS scores and BATS scores at baseline, fourth, and eighth session for the individual therapy and group setting combined. *p < 0.05 significant score reductions from baseline to eighth session

Premonitory urge (evaluated by PUTS) and belief about the tics (evaluated by BATS) were measured at baseline, session 4, and session 8. There was no significant change in the total PUTS scores during treatment (Fig. 3). The PUTS score correlated with the assessment of the global premonitory urge (at baseline: rho 0.78 (0.25–1.30), *p* = 0.005, and at session 8: rho 0.87 (0.27–1.46), *p* = 0.005).

The BATS scale showed a significant total reduction of 7.74 (4.79–10.70) (*p* = 0.0001) point score which was significantly larger in the individual group compared to group treatment (*p* = 0.0075) (Fig. 3). The PUTS scale and the BATS scale were positively associated both at baseline [rho 0.22 (0.07–0.38), *p* = 0.005], at session 4 [rho 0.40 (0.26–0.53), *p* = 0.0001], and at session 8 [rho 0.31 (0.18–0.44), *p* = 0.0001].

Parents reported a significant reduction in scores measured on SCARED (*p* = 0.0019), which could not be shown in the children’s ratings. There were no between-group differences. No differences could be shown for MFQ scores.

*N* = 9 (15.3%) of the participants reported that they had preferred HRT compared to *N* = 19 (32.2%) who had preferred ERP, and *N* = 16 (27.1%) reported the use of both methods. A subjective feeling of having control (*N* = 36) or a reduced tic intensity (*N* = 30) was the most often reported effects of the training program.

## Discussion

This is the first Scandinavian study evaluating the effectiveness of a newly developed manual combining ERP and HRT [24]. To our knowledge, it is also the first randomized controlled study that compares the efficacy of therapeutic treatments based on the same manual delivered individually or in a group setting.

Compared to other studies, the present study shows that treatment combining HRT and ERT training is effective in both groups and as an individual treatment. The decrease from baseline to end point on the Total Tic score (motor and vocal tic score) of YGTSS of 9.48 points (individually) and 7.48 points (group setting) (8.48 points for all participants) is comparable or slightly greater than the effects shown in previous studies [13, 19]. There was no significant difference in Total Tic score between individual and group treatment. Children and adolescents were trained in both HRT and ERP, which gave them a possibility to alternate between the strategies depending on their general situation. In both the group setting and in individual therapy, a substantial number of the participants described that they used a combination of the methods using HRT for certain selected tics and ERP for training against all tics. Defining a 25% reduction on the YGTSS Total Tic score as predictive for a positive response [38], 66.7% of the participants were rated as responders. Verdellen et al. described the percentage of patients who improved more than 30% [19]. They showed that 58% of patients in the ER group and 28% in the HR showed a reduction that exceeded 30%. In the present study, a reduction of more than 30% was shown for 59.3% of participants.

The participants reported a significant reduction in the functional impairment score, which was significantly greater in the individual setting. Also, measured by the subjective global scores, the individual training showed the greatest outcomes. In an individual setting, the interaction between the therapist and the child may become more immediate, and the therapist is able to focus more intensely on a particular child’s resources and difficulties. Furthermore, the parents may experience a better opportunity to discuss the influence on the family. These circumstances may influence the general functional impairment experienced by both the child and the family. For both the individual and groups setting, most of the participants reported to have experienced achieving a subjective feeling of having control or reduced tics intensity. Thus, several participants reported that they might still have tics, but that the tics had become less restrictive for their lives. The finding that group HRT/ERP is an effective treatment validates the only other study that examines the effect of group treatment (HRT versus educational treatment [21]).

Sensory phenomena are very frequent in TS, premonitory urges being one of the most often reported preceding sensations [36]. In children, however, a developmental aspect has to be considered since younger children rarely experience a premonitory urge [39]. In the present study, PUTS scores showed no difference from baseline to session 8 even though tic severity was reduced. In a study from 2013, Specht et al. showed that urge ratings did not show an increase during the initial periods of tic suppression, or a decline in urge intensity during the following prolonged tic suppression [40]. Correspondingly, Ganos et al. found no correlation between scores of premonitory urges and the ability to suppress tics [13]. Furthermore, the premonitory urge has been shown to remain unchanged during tic suppression [12, 41]. Thus, some patients may not experience a habituation to the premonitory urge, but rather have to learn to accept and endure the urge feeling. There was no difference in change of PUTS scores between individual and group setting. The PUTS score correlated at all time points with the BATS score. The BATS score was reduced significantly from treatment start to end point, suggesting that tics treatment had a significant impact on thoughts and interpretations of tics. Furthermore, the scores of the BATS were significantly more reduced during the individual therapy compared to the group setting, suggesting that individual treatment is more likely to have an indirect effect on the children’s interpretations of their tic disorder. To our knowledge, this is the first study comparing the influence of group or individual treatment on BATS scores in children and adolescents with Tourette syndrome.

Parents rated a significant decrease in anxiety scores measured by SCARED, whereas the children did not report a change. Similarly, no change was reported on the MFQ scale. There were no in-between-group differences.

Our results have several clinical implications. First, the efficacy of a combined treatment of HRT and ERP in both an individual and group setting expands the available treatment possibilities for tic disorders in children and adolescents. The participants represented a clinical sample with few exclusion criteria; thus the manual has a broad applicability. The efficacy shown in the present study is comparable with those found in medication treatment studies [42, 43], further increasing the argument for a therapeutic treatment in preference to medication. Secondly, the children achieved an experience of enhanced control of their tic disorder, and the study shows an important influence on the interpretation and beliefs in relation to tic symptoms rather than merely an effect on tic severity.

There are limitations to the trial. There was no control group to control for the natural course of TS and with regard to treatment. The present study aimed at comparing the outcome of individual therapy to the outcome of group therapy. The methods that were combined in the present study, HRT and ERP, are well-established treatment methods in an individual setting with effect sizes comparable to those presented in the present study. Thus, even though effect sizes for the combined treatment are comparable with effect sizes for either HRT or ERP, the combination renders the child able to choose the suitable method depending on the tic symptom and their general situation. The number of included patients was small, although sufficiently large to show a significant reduction in Total Tic score measured on the YGTSS. However, a higher number may be needed to detect between-group differences. The present study included only acute outcome data. Further research into the durability of the treatment effect is warranted.

## Abbreviations

TS: Tourette syndrome
HRT: Habit reversal training
ERP: Exposure response prevention
CBIT: Comprehensive behavioral intervention for tics
ADHD: Attention deficit hyperactivity disorder
K-SADS-PL: Schedule for Affective Disorders and Schizophrenia for School-Age Children—Present and Lifetime version
CBCL: Child Behavior Checklist
SDQ: Strengths and Difficulties
SP: Sensory profile
YGTSS: Yale Global Tics Severity Scale
SCARED: Screen for Child Anxiety Related Emotional
MFQ: The Mood and Feelings Questionnaire
PUTS: Premonitory Urge Scale
BATS: Beliefs About Tics Scale
